# No Decrease in Muscle Strength after Botulinum Neurotoxin-A Injection in Children with Cerebral Palsy

**DOI:** 10.3389/fnhum.2016.00506

**Published:** 2016-10-06

**Authors:** Meta N. Eek, Kate Himmelmann

**Affiliations:** Department of Pediatrics, Institute of Clinical Sciences, The Sahlgrenska Academy, University of GothenburgGothenburg, Sweden

**Keywords:** cerebral palsy, spasticity, botulinum toxin, muscle strength, children

## Abstract

Spasticity and muscle weakness is common in children with cerebral palsy (CP). Spasticity can be treated with botulinum neurotoxin-A (BoNT-A), but this drug has also been reported to induce muscle weakness. Our purpose was to describe the effect on muscle strength in the lower extremities after BoNT-A injections in children with CP. A secondary aim was to relate the effect of BoNT-A to gait pattern and range of motion. Twenty children with spastic CP were included in the study, 8 girls and 12 boys (mean age 7.7 years). All were able to walk without support, but with increased muscle tone interfering with motor function and gait pattern. Sixteen children had unilateral spastic CP and four bilateral spastic CP. Twenty-four legs received injections with BoNT-A in the plantar flexor muscles. The children were tested before treatment, around 6 weeks after at the peak effect of BoNT-A, and at 6 months after treatment, with measurement of muscle strength, gait analysis, and range of motion. There were no differences in muscle strength in plantar flexors of treated legs at peak effect compared to baseline. Six months after treatment, there was still no change in untreated plantar flexor muscles, but an increasing trend in plantar flexor strength in legs treated with BoNT-A. Parents reported positive effects in all children, graded as: small in three children, moderate in eight, and large in nine children. The gait analysis showed a small improvement in knee extension at initial contact, and there was a small increase in passive range of motion for ankle dorsiflexion. Two children had a period with transient weakness and pain. We found that voluntary force production in plantar flexor muscles did not decrease after BoNT-A, instead there was a trend to increased muscle strength at follow-up. The increase may be explained as an effect of the blocking of involuntary nerve impulses, leading to an opportunity to using and training the muscles with voluntary control. Adequate muscle strength is important for maintaining the ability to walk and knowledge of how a treatment affects muscle strength is useful when selecting interventions.

## Introduction

Spasticity is a common problem in children with cerebral palsy (CP) (SCPE, 2002; Himmelmann et al., 2007), which may have an adverse effect on motor development (Gormley, 2001). About 80% of CP is classified as spastic. Spasticity can be defined as a velocity-dependent increase in resistance to passive movement (Sanger et al., 2003), which may prevent voluntary movement and cause muscle shortening (contracture), which in turn can also affect skeletal development.

It has been possible since the 1990s to treat spasticity with botulinum neurotoxin A (BoNT-A). BoNT-A is injected directly into the muscle and inhibits the release of neurotransmitter at the neuromuscular junction, thus blocking nerve impulses to the muscle (Simpson et al., 2008). The effect will increase gradually over a few weeks and maximum effect is usually seen after four to 6 weeks, when the muscle becomes more flexible. The effect is reversible. Improving gait in children who can walk but in whom spasticity is an obstacle is one indication.

The muscle is theoretically weakened by blocking nerve impulses to the muscle fibers, and muscle weakness has been reported in experiments with rabbits and cats (Yaraskavitch et al., 2008; Fortuna et al., 2011). In studies of BoNT-A treatment in children with CP, muscle weakness also has been reported, although only often as comments (Naumann et al., 2006; O’Flaherty et al., 2011). Before the start of the present study we found only one study that actually measured muscle strength (Bjornson et al., 2007). Bjornson et al. compared BoNT-A to a control group receiving saline injections and found that muscle strength had increased 24 weeks after treatment in the BoNT-A group but not in the group receiving saline injections. Thus the reports are conflicting and it is not fully known how BoNT-A affects voluntary muscle strength.

Muscle weakness is common in children with CP and has been described as being more pronounced distally and with an imbalance across joints (Wiley and Damiano, 1998; Ross and Engsberg, 2002). It has been shown to correlate with walking ability, where more than 50% of the predicted normal strength was associated with the ability to walk without aids (Eek and Beckung, 2008). A relationship between muscle strength and gait has been demonstrated in terms of velocity, stride length, gait kinematics, and the gross motor function measure (GMFM; Damiano et al., 2000; Desloovere et al., 2006; Ross and Engsberg, 2007). Gage described five prerequisites for a normal gait pattern (Gage, 2004); stability in stance, foot clearance in swing, pre-positioning of the foot for initial contact, adequate step length, and energy conservation, all of which may be compromised by muscle weakness. The plantar flexor muscles are of special interest as they are the major force generators for forward progression (Kepple et al., 1997; Sadeghi et al., 2001).

Weakness often co-exists with spasticity. This is almost always the case in the gastrocnemius muscle in children with CP. However, muscle strength and selective motor control have a higher correlation with gait parameters than spasticity and joint mobility (Ross and Engsberg, 2002; Ostensjo et al., 2004; Desloovere et al., 2006). With this in mind, it is important not to make weak muscles even weaker with treatment, with a risk of compromising gait pattern and walking ability.

The plantar flexor muscle group is difficult to measure in typically developing children above the age of nine because of the short lever arm, leading to high forces and difficulties in stabilizing with a hand held device (Eek et al., 2006). However, as children with CP often exhibit weakness in the plantar flexors, this makes it possible to measure muscle strength among children over 9 years of age as well.

The primary aim of the study was to describe the effect on muscle strength in the lower extremities after BoNT-A injections in children with CP by means of measurement of torque. A secondary aim was to relate the effect of BoNT-A to gait pattern and joint range of motion.

## Materials and Methods

### Participants

Children with spastic CP were recruited consecutively from the spasticity clinic at the Regional Rehabilitation Centre in Gothenburg, Sweden. Inclusion criteria were increased muscle tone interfering with motor function/gait pattern and ability to walk without support. Twenty-three children were recruited; three were lost to follow-up (due to not returning for follow-up), resulting in a total of 20 children, 8 girls and 12 boys with a mean age of 7 years 8 months at injection (*SD* 2:9). Sixteen children had unilateral spastic CP and four bilateral spastic CP, according to the CP classification by Surveillance of Cerebral Palsy in Europe (SCPE, 2000). Nineteen children were classified at level I, according to the gross motor function classification system (GMFCS) and one with bilateral spastic CP at level II (Palisano et al., 2006). Their mean weight was 27.3 kg (*SD* 11.7) and mean height 125.5 (*SD* 17.4).

### Procedure

The children were tested three times: at baseline before treatment with BoNT-A, at peak effect around 6 weeks after, and at a follow-up when the effect leveled off around 24 weeks after injection.

BoNT-A injections (Botox^®^; Allergan or Dysport^®^, Ipsen) were made during sedation with ketamine and midazolam in combination (rectal administration), or with nitrous oxide sedation, after local anesthetic EMLA^®^ cream was applied to injection sites. Injections were guided with neuromuscular electric stimulation; using Teflon-coated Botox needles (37 mm, 27 gage) diluted to100 Units (U)/mL. Doses were calculated according to size of the muscle, body weight, and degree of spasticity (Love et al., 2010).

Measurements of muscle strength were made in four muscle groups (knee: extensors and flexors, ankle: dorsi- and plantar flexors) with a handheld device (Chatillon, AMETEK Test & Calibration Instruments, USA) using the “make” technique and standardized positions. The positions used in this study were similar to a normative study (Eek et al., 2006). Three attempts were made after instruction and familiarization with the procedure, and the maximum recording was used for data analysis. The lever arm for each muscle group was measured with a tape measure, and torque was calculated and normalized to body weight (Nm/kg). Muscle strength in children with CP can be measured with good reliability with a handheld electronic myometer (Berry et al., 2004; Taylor et al., 2004).

A two-dimensional gait analysis was performed in all legs with BoNT-A injections in the gastrocnemius muscle, with registration of spatiotemporal data and joint kinematics. For data acquisition, we used a motion capture camera with a sampling rate of 50 Hz (Oqus, Qualisys AB, Göteborg, Sweden), placed at a distance of 3 m from the subject. Four markers (∅ 16 mm) were attached with double-sided adhesive tape to the skin at bony landmarks (trochanter major, lateral knee joint, lateral malleolus, and head of metatarsale V). Data was processed with the QTM software (Qualisys AB, Göteborg, Sweden) and exported to an excel worksheet for calculation of gait velocity, stride length, and joint angles in the knee and ankle, using the coordinates of the reflective markers. The same examiner, who has 15 years of experience with the method, placed the markers. Children walked barefoot, and measurements where parents confirmed that the gait pattern was relaxed and typical for the child were chosen for analysis. Values for joint angles at initial contact, as well as peak values for knee extension and dorsiflexion in stance were used for statistical testing.

Passive range of motion (ROM) was measured with a plastic goniometer. Spasticity was graded according to a modified Ashworth scale (Bohannon and Smith, 1987) with the children lying supine in a relaxed position with head in midline.

The children received physiotherapy during 2–3 months post injection with their regular therapist, with individual programs focusing on active movements and selective control around the ankle, as well as activities including gait and balance.

One month after injection, parents were interviewed or asked to fill in a questionnaire about the effect of the treatment. This was reported as a positive and/or negative effect and graded as: no effect, small effect, moderate effect or large effect.

This study was approved by the Regional Ethics Review Board, University of Gothenburg, with written informed consent from parents of all subjects.

### Statistical Analysis

According to a previous study on muscle strength training in children with CP, a sample size of 17 was sufficient to analyze change in muscle strength with a power of 0.8 (Eek et al., 2008).

Muscle strength in the plantar flexor group was analyzed in children given injections in the gastrocnemius muscles, and knee flexor strength was analyzed for injections in the hamstring muscles. Muscle strength in the corresponding muscle groups in untreated legs served as controls. Muscle strength in the antagonists (ankle dorsiflexors and knee extensors) was also used for comparison.

Data were tested for normality with the Shapiro–Wilks test. It was found that all variables were not normally distributed, and non-parametric methods were thus used for analysis. Data were analyzed with the Mann–Whitney *U*-test for comparison between treated and control legs at baseline. Repeated measures ANOVA was used for comparison before and after treatment, and with a post hoc test with Bonferroni correction for multiple comparisons, for variables showing a statistically significant difference in the ANOVA test. When assumption of sphericity was violated, the Greenhouse–Geisser correction was used. The software packages Statview and SPSS were used for the analyses. A *p*-value less than 0.05 was considered statistically significant.

## Results

Both legs were treated in children with bilateral involvement; in total the gastrocnemius muscles of 24 legs/20 children received injections with BoNT-A. In addition the soleus muscles were treated in 9 legs/9 children and m. tibialis posterior in 10 legs/8 children. Eight legs/six children had injections in hamstring muscles. Mean dose/kg bodyweight injected into gastrocnemius was (Botox^®^) 3.2 Allergan units/kg, or (Dysport^®^) 6 units/kg; mean dose in hamstrings (Botox^®^) 1.5 Allergan units/kg, or (Dysport^®^) 2.5 units/kg.

Three children wore lower limb casts after the injection; one child wore a cast for 2 weeks and two children for 2+2 weeks. The casts were put on 2 weeks after treatment to allow time for muscle relaxation before the children were given the casts. Measurements of muscle strength for these children were made at least 2 weeks after the casts were removed.

Tests at peak effect were made at a mean of 5 weeks and 5 days (*SD* 1 week 3 days) after injection and were followed up at 5 months and 3 weeks (*SD* 1 month) after.

### Muscle Strength

**FIGURE 1:**
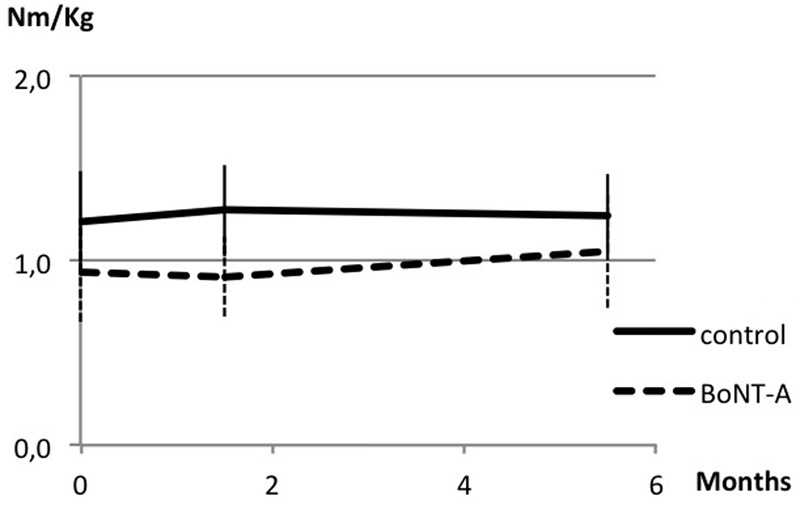
**Muscle strength in plantar flexor muscles at baseline, peak effect and follow-up after botulinum neurotoxin-A (BoNT-A) treatment.** Data in torque normalized to body weight (Nm/kg), presented as mean and with SD error bars.

**FIGURE 2:**
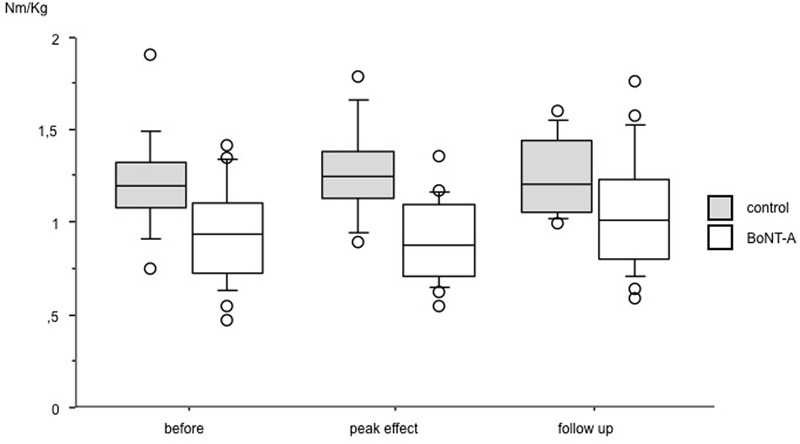
**Muscle strength in plantar flexor muscles at baseline, peak effect and follow-up after BoNT-A treatment.** Data in torque normalized to body weight (Nm/kg), presented as boxes indicating 25–75 percentile, a line indicating median and circles showing observations outside 10 or 90 percentile.

Data on muscle strength are presented in **Table 1** and **Figures 1** and **2**. At baseline, plantar flexor muscles subjected to BoNT-A were weaker than control muscles (*p* = *0.005*, confidence interval (CI) 0.10; 0.44). There was no difference in plantar flexor strength at peak toxin effect as compared to baseline. At follow-up 6 months after treatment, repeated measures ANOVA showed no difference in control muscles, but a difference in plantar flexor muscles with BoNT-A injection [*F*(2,46) = 4.44, *p = 0.017*], showing a trend to increased plantar flexor strength (CI -0.25; -0.01). The opposite pattern was found in the knee flexor group, with no change in knee flexors treated with BoNT-A but an increase in control muscles [*F*(1.7,52.3) = 4.60, *p = 0.019*] (CI -0.14; -0.02). In the antagonistic muscle groups, there was no change of strength in dorsiflexors, but increased muscle strength in knee extensors [F(2,78) = 8.38, *p = 0.001*] (CI -0.31; -0.07).

**Table 1:** Muscle strength, gait data and range of motion at baseline, peak effect and follow-up, presented as median (interquartile range).

			Baseline	Peak effect	Follow up	*ANOVA*	*diff 1-2*	*diff 1-3*
**Muscle strength Nm/kg**		***n***						***p***

Plantar flexors	BoNT-A	24	0.94 (0.38)^†^	0.88 (0.38)	1.02 (0.43)	***0.017***	*1.000*	*0.069*
	control	14	1.20 (0.25)^†^	1.25 (0.25)	1.20 (0.39)	*0.660*		
Knee flexors	BoNT-A	8	0.91 (0.33)	0.89 (0.43)	0.89 (0.35)	*0.445*		
	control	32	0.90 (0.29)	1.04 (0.29)	1.00 (0.34)	***0.019***	***0.036***	***0.008***
Dorsiflexors		40	0.31 (0.15)	0.32 (0.17)	0.34 (0.20)	*0.916*		
Knee extensors		40	1.37 (0.52)	1.52 (0.69)	1.48 (0.75)	***0.001***	*0.180*	***0.001***

**Gait data for legs with gastrocnemius injections**					

Velocity m/sec		20	1.06 (0.18)	1.06 (0.18)	1.11 (0.19)	*0.159*		
Stride length		20	0.98 (0.24)	0.98 (0.18)	1.04 (0.27)	*0.060*		
Knee angle at initial contact (°)		22	19.8 (14.9)	17.7 (9.6)	20.7 (14.4)	***0.031***	***0.035***	*0.469*
Knee extension in stance (°)		23	11.8 (11.8)	11.4 (9.9)	10.2 (9.6)	*0.100*		
Ankle angle at initial contact (°)		23	-7.0 (13.9)	-7.9 12.4)	-8.3 (9.9)	*0.414*		
Dorsiflexion in stance (°)		23	8.2 (7.4)	9.5 (8.5)	6.6 (4.4)	*0.619*		

**Range of motion**					

Passive dorsiflexion with extended knee (°)		15	15.0 (8.8)	15.0 (9.5)	18.0 (11.0)	***0.048***	*0.058*	*0.114*

*p-values (*in italic*) for ANOVA and at peak effect and follow up compared to measurement at baseline for variables with a statistically significant ANOVA test. Statistically significant differences indicated in bold figures. ^†^Statistically significant difference between muscles treated with botulinum neurotoxin-A (BoNT-A) and control muscles at baseline *p* = 0.004.*

### Gait

There was no difference in gait velocity or stride length after treatment.

Kinematic gait data from all three occasions were available in 23 legs/20 children with BoNT-A treatment in the gastrocnemius. At peak effect, the 2D gait analysis showed a small improvement in knee extension at initial contact [*F*(2,44) = 3.78, *p = 0.031*] (CI 1.1; 6.5). At baseline the ankle was in plantar flexion at initial contact in all but two legs subject to BoNT-A treatment. There were no differences regarding ankle angle in gait after treatment.

### Muscle Tone and Passive ROM

Spasticity grading of the gastrocnemius muscle at baseline was available in 35 legs (23 with BoNT-A and 12 controls). Muscle tone at baseline was increased in legs planned for treatment (grade 1 in 13, grade 1+ in 6, and grade 2 in 4) and in five control legs (grade 1). Grading of spasticity after treatment was available in 14 of the legs treated with BoNT-A. There was a decrease in muscle tone in 7 legs and no change in the rest.

Data on passive dorsiflexion in the ankle at baseline were available in 31 legs (19 BoNT-A and 12 controls). There was a statistically significant difference (*p* < 0.001, CI 7.8; 18.1) between muscles planned for injection (median 15.0°, interquartile range (IQR) 8.8) compared to controls (median 22.5°, IQR 11.0). Data from all three occasions were available in 15 legs treated with BoNT-A in the gastrocnemius (**Table 1**). There was a small increase in passive dorsiflexion [*F*(2,28) = 3.40, *p = 0.048*].

### Parent Reports

The parent reports all indicated positive effects graded as: small (three children), moderate (eight children), and large (nine children). Some parents specifically mentioned that their child was not walking on toe and could more easily make heel contact. One child had pain for a couple of weeks after the casts, and one child felt insecure/weak in the first weeks after injection.

## Discussion

The primary aim of the study was to see whether voluntary muscle strength is affected by BoNT-A, with a focus on plantar flexor muscles, as being important for walking. We found no sign of decreased torque in plantar flexor muscles at the point at which the toxin effect was at its peak. There was instead an increasing trend in strength at follow-up in muscles treated with BoNT-A. No increase was seen in the untreated plantar flexors that acted as controls during the study period. This finding is similar to that in a study by Bjornson et al. (2007) who found increased muscle strength 6 months after treatment compared to saline injections. Williams et al. (2013b) found no difference in strength 5 weeks after injections in the gastrocnemius, while Barber et al. (2013) found an increased muscle volume after BoNT-A treatment, suggesting increased muscle strength. In reports of adverse effects, weakness is mentioned as occurring over short transient periods during the first weeks after injection (Naumann et al., 2006; O’Flaherty et al., 2011). The parents of one child in our study reported a transient period of muscle weakness in the first days/first week after injection.

It has been demonstrated that the concurrent treatment of BoNT-A and strength training can achieve positive outcomes in terms of strength, spasticity and for the achievement of functional goals (Williams et al., 2013a). Physiotherapy after injection in our study focused on voluntary control in the ankle, and exercises in standing balance and gait. Many of these activities, as well as everyday life, include using both legs, and both legs thus receiving about the same amount of muscle activity. In our study, most of the children had injections in only one leg, and in this way muscle strength development in not injected muscles in the other leg could serve as control to injected muscles. Spasticity, muscle weakness, and poor voluntary control often co-exist in children with CP (Gormley, 2001). The increasing trend in muscle strength in plantar flexors after BoNT-A can be explained as an effect of the blocking of involuntary nerve impulses leading to an opportunity to take better voluntary control, and the increasing trend in muscle strength as an effect of training and using the muscle with voluntary control. As standing and gait involves muscle activity in the whole leg it may not be surprising that there was also increased muscle strength in knee extensors and knee flexors. Contrary to this we found no change in ankle dorsiflexors. In growing children, 6 months may be sufficient time for a natural increase in muscle strength and here the not injected muscles also can serve as controls. According to a previous study of muscle strength in children with CP, 5-15 years of age (Eek, 2009), there is an increase in torque with age for knee extensors and knee flexors, but not for ankle plantar and dorsiflexors. The findings in the present study can therefore be interpreted as an expected development for knee extensors, knee flexors and ankle dorsiflexors.

Gait pattern improved regarding knee extension at initial contact in legs with BoNT-A treatment in the gastrocnemius, but there were no changes in ankle angle in the gait analysis. This is different from previously reported findings of improved peak ankle dorsiflexion in stance after BoNT-A (Sutherland et al., 1999). Interestingly, parents reported improvement in heel strike and foot contact, which is similar to a study with video analysis, who found improvement in initial foot contact following BoNT-A (Ubhi et al., 2000). However, an improved position in the knee at initial contact and during the stance phase facilitates the possibility to make heel contact (which is easy to see with the eye) while the exact angle in the knee and ankle is difficult to see without instrumented measurement. In contrast to the lack of improved dorsiflexion in gait, there was increased passive ROM in the ankle which was small but statistically significant. Even small differences can be clinically relevant if the increase in dorsiflexion is close to the neutral position of the ankle, and letting the ankle dorsiflex a few degrees can be what is needed for a better gait pattern. The lack of change in ankle angle in the gait pattern may be explained by weakness in dorsiflexors, and also the fact that as a bi-articular muscle the improved range of motion in gastrocnemius was gained at the proximal end, thus facilitating knee extension.

The children in the study did not have very severe spasticity, most of them with a grade 1–1+ according to the Ashworth scale, with a resistance to quick stretch at the end or in the second half of the movement. There was decreased spasticity in only half of the treated legs after treatment. This may depend on the Ashworth scale as a subjective way of testing and also that muscle tone may vary with the situation, and if the child is excited. Testing was, however, performed in a standardized way with the child in rest, lying supine with the head in midline. This position does not always reflect what is happening in activity and at baseline assessment it was visible that there was an increase in muscle tone in activity, affecting movement patterns. There is yet no way (that we know of) of measuring muscle tone in activity in a clinical setting.

### Limitations

There are some limitations that need to be acknowledged in this study. There was a variety in location of injections; some children were also given injections in soleus, tibialis posterior, and hamstring muscles in addition to gastrocnemius. This makes the sample heterogeneous but they were considered too few to be possible to analyze as subgroups in this study. Three children were immobilized in the ankle by casts for 2 weeks after BoNT-A, to increase stretch. This immobilization can induce weakness and for that reason measurement of muscle strength was performed at least 2 weeks after removal of the casts, to let them regain some muscle strength.

However, the primary aim and focus of the study was to measure whether muscle strength deteriorated, and we found that this was not the case. There was instead an increasing trend in muscle strength at follow-up.

### Clinical Implications

Voluntary force production in plantar flexor muscles was not decreased by BoNT-A. Muscle strength instead showed an increasing trend at follow-up. Gait pattern improved in terms of better knee extension in stance.

Adequate muscle strength is important for maintaining the ability to walk. Knowledge of how a treatment affects muscle strength is necessary when selecting interventions in order not to make muscles weaker with treatment.

## Author Contributions

ME and KH made substantial contributions to the design, acquisition, analysis, and interpretation of data for the work, as well as drafting and final approval of the version to be published. And agree to be accountable for all aspects of the work in ensuring that questions related to the accuracy or integrity of any part of the work are appropriately investigated and resolved.

## Conflict of Interest Statement

The authors declare that the research was conducted in the absence of any commercial or financial relationships that could be construed as a potential conflict of interest.
